# The origin of the octoploid cloudberry (*Rubus chamaemorus*) genome is the result of multiple and complex polyploidization events

**DOI:** 10.1093/jhered/esag028

**Published:** 2026-03-25

**Authors:** Marius A Strand, Thu-Hien To, Ole K Tørresen, Sarah Fullmer, Giada Ferrari, Morten Skage, Ave Tooming-Klunderud, Simen Rød Sandve, Kjetill S Jakobsen

**Affiliations:** Department of Biosciences, Centre for Ecological and Evolutionary Synthesis (CEES), University of Oslo, Oslo, Norway; Faculty of Biosciences, Department of Animal and Aquacultural Sciences, Center for Integrative Genetics (CIGENE), Norwegian University of Life Sciences, Ås, Norway; Faculty of Biosciences, Department of Animal and Aquacultural Sciences, Center for Integrative Genetics (CIGENE), Norwegian University of Life Sciences, Ås, Norway; Department of Biosciences, Centre for Ecological and Evolutionary Synthesis (CEES), University of Oslo, Oslo, Norway; Department of Biosciences, Centre for Ecological and Evolutionary Synthesis (CEES), University of Oslo, Oslo, Norway; Department of Biosciences, Centre for Ecological and Evolutionary Synthesis (CEES), University of Oslo, Oslo, Norway; Department of Biosciences, Centre for Ecological and Evolutionary Synthesis (CEES), University of Oslo, Oslo, Norway; Department of Biosciences, Centre for Ecological and Evolutionary Synthesis (CEES), University of Oslo, Oslo, Norway; Faculty of Biosciences, Department of Animal and Aquacultural Sciences, Center for Integrative Genetics (CIGENE), Norwegian University of Life Sciences, Ås, Norway; Department of Biosciences, Centre for Ecological and Evolutionary Synthesis (CEES), University of Oslo, Oslo, Norway

**Keywords:** allopolyploidization, autopolyploidization, Earth BioGenome Project Norway, haplotype-resolved, HiFi sequencing, octoploid

## Abstract

We describe a chromosome-level genome assembly from an individual male plant of the cloudberry (*Rubus chamaemorus*). The haplotype-resolved assemblies contain 1 pseudo-haplotype spanning 1,198 megabases and 1 pseudo-haplotype spanning 1161 megabases. Most of these 2 assemblies, 93.57% and 96.55% respectively, are each scaffolded into 28 pseudo-chromosomes. Both assemblies show high completeness, with the same Benchmarking Universal Single-Copy Orthologs (BUSCO) completeness score of 99.2%. Most BUSCO genes are duplicated in both pseudo-haplotypes, in line with the polyploid nature of the cloudberry genome. The assemblies contain 74,132 and 70,692 predicted protein-coding genes, respectively. Analysis of repetitive sequences classified ~60% of each haplotype as repeats. Comparative synteny with red raspberry (*Rubus idaeus*) reveals a 4:1 chromosome correspondence, supporting an octoploid origin. Across k-mer composition, synonymous divergence, and genome-wide gene-tree analyses, 1 set of 7 chromosomes is consistently distinct (β), while the remaining 21 (3 × 7) chromosomes form an α set with no resolvable internal subdivision. These results are consistent with a complex polyploid origin of the cloudberry genome, involving a combination of allopolyploid and autopolyploid processes.

**Significance statement**

Cloudberry cultivation has lagged due to limited genomic resources and an unresolved polyploid history. We present a haplotype-resolved, chromosome-scale assembly that resolves 4 homologs per ancestral chromosome into 28 pseudo-chromosomes per haplotype and shows a 4:1 correspondence with red raspberry (*Rubus idaeus*). Genome-wide analyses support an octoploid architecture with 1 distinct 7-chromosome set (β) and a remaining α-set of 21 chromosomes with no resolvable internal subdivision. This reference provides a foundation for future work on polyploid origin, trait discovery, and breeding.

## Introduction

**Fig. 1:**
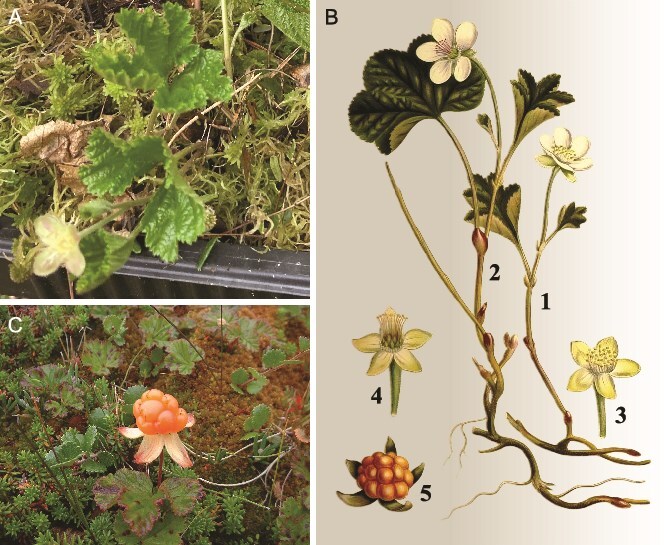
Illustration of dioecy in *R. chamaemorus* and the sequenced male specimen. *Rubus chamaemorus* is dioecious, with male and female flowers borne on separate plants. A) Our sample (a male plant) photographed late in the season (photo: Simen R. Sandve). B) Historical illustration: (1) male and (2) female plant in bloom; (3) male and (4) female flowers with petals removed; (5) calyx and aggregate fruit (adapted from *Bilder ur Nordens Flora*, author: C.A.M. Lindman, via https://runeberg.org/nordflor/311.html, public domain). C) Female plant with ripe aggregate fruit (image from Wikimedia commons, author: Philipum, public domain).

The cloudberry (*Rubus chamaemorus*) is a circumpolar boreal flowering plant species in the rose family (Urbaniak et al. 2023). Within the rose family (subfamily Rosoideae) *Rubus* is a diverse genus with an estimated number of species ranging from 250 to 1,000 (Huang et al. 2023) found on all continents except Antarctica. Many species of *Rubus* are known for their aggregate fruits, such as blackberries, raspberries, dewberries, and several hybrids. Aggregate fruits of *R. chamaemorus* are golden-yellow, soft and juicy, rich in vitamin C, ellagic acid, citric acid, and anthocyanins, giving it a distinctive tart taste (see Fig. 1). They are used in jams, desserts, and liqueurs, and are considered a delicacy, particularly in Nordic countries. Despite great demand, *R. chamaemorus* is not commonly cultivated and is primarily harvested as a wild plant.

Unlike most *Rubus* species, *R. chamaemorus* has biparental (dioecious) reproduction with distinct unisexual individuals, each producing either male or female gametes (see Fig. 1B). Dioecy is a strategy to avoid self-fertilization and promote outcrossing. Within *Rubus,* around 60% to 70% of the species are polyploid, ranging from 4x up to 14x (Thompson 1997; Hummer et al. 2016). *Rubus chamaemorus* itself is a known octoploid with 8 genome copies. Evidence from nuclear and chloroplast phylogenies suggests a complex hybrid origin (Michael 2006; Carter et al. 2019). Michael (2006) found chloroplast DNA support for *Rubus pedatus* as the maternal ancestor, and nuclear GBSSI-1γ sequences in *R. chamaemorus* revealed 2 divergent copies: one clustering with *Rubus lasiococcus*, the other with *Rubus arcticus*. However, based on internal transcribed spacer sequences, *R. arcticus* was rejected as a possible progenitor. This is consistent with Carter et al. (2019), who placed *R. chamaemorus* in an early-diverging lineage outside the main *Rubus* clade. In addition to interest in its complex polyploidy, *R. chamaemorus* has also received interest for potential cultivar development (Martinussen et al. 2013), as well as regional conservation efforts (Urbaniak et al. 2023).

**Fig. 2:**
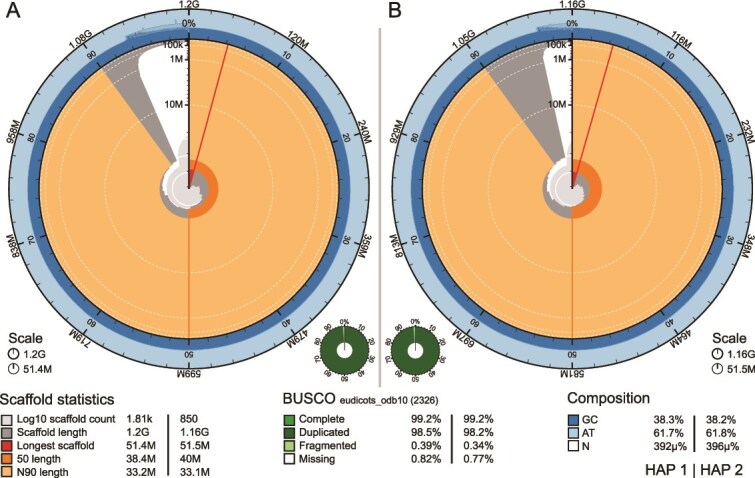
Metrics of the genome assemblies of *R. chamaemorus* hap1 and hap2. A) Hap1. B) Hap2. The BlobToolKit Snailplots show N50 metrics and BUSCO gene completeness. The 2 outermost bands of the circle signify GC vs. AT composition at 0.1% intervals. Inner plot features show the N90 scaffold length (light orange), N50 scaffold length (dark orange), and the largest scaffold (red). All the scaffolds are arranged in a clockwise manner from the largest to the smallest and are shown in darker gray with white lines at different orders of magnitude, while the light gray shows cumulative count of scaffolds.

Despite the benefit for potential cultivar development, no genome assembly has been made available to date. Here, we have used long-read HiFi (PacBio) sequencing combined with long-range chromosomal contact mapping by Hi-C to assemble the first high-quality, haplotype-resolved, chromosome-scale genome of *R. chamaemorus* (Fig. 2). The resulting genome consists of 2 haplotype-resolved assemblies spanning 1,198 and 1,161 Mb, respectively, with 93.57% and 96.55% of the estimated genome scaffolded into 28 pseudo-chromosomes. Gene annotation identified 74,132 and 70,692 protein-coding genes, respectively. Comparison with other available *Rubus* genomes indicates that the octoploid architecture of *R. chamaemorus* reflects a complex polyploid origin involving both allopolyploid and autopolyploid processes. The 2 haplotype-resolved assemblies provide a genomic foundation for future genetic, evolutionary, and comparative studies of *R. chamaemorus*.

## Results

### De novo genome assembly and annotation

The genome from an individual male *R. chamaemorus* plant was assembled from a total of 59-fold coverage in Pacific Biosciences single-molecule HiFi long reads and 62-fold coverage in Arima Hi-C reads, resulting in 2 haplotype-separated assemblies. The final assemblies have total lengths of 1,198 and 1,161 Mb (Table 1 and Fig. 2), respectively. Pseudo-haplotypes 1 (hap1) and 2 (hap2) have scaffold N50 size of 38.4 and 40.0 Mb, respectively, and contig N50 of 29.4 and 33.1 Mb, respectively (Table 1 and Fig. 2). Twenty-eight pseudo-chromosomes were identified in both pseudo-haplotypes (ordered by homology to *Rubus idaeus* chromosomes first, and length in hap1 second, and then numbered 1 to 28, with the homologs in hap2 receiving the same number).

**Table 1:** Genome data for *R. chamaemorus.*

**Project accession data**		
Species	*Rubus chamaemorus*	
Specimen	drRubCham1	
NCBI Taxonomy ID	57,936	
BioProject	PRJEB98354	
BioSample ID	SAMEA120268085	
Isolate information	Male, leaf	
**Raw data accessions**		
PacBio HiFi reads	ERR15658311, ERR15658288	2 PACBIO_SMRT (Sequel IIe) runs: 4.66 M reads, 70.8 Gb
Hi-C Illumina reads	ERR15658302	1 ILLUMINA (Illumina NovaSeq 6000) run: 246 M pairs of reads, 74.3 Gb
**Genome assembly metrics**		
HiFi read coverage	59x	
Assembly accession	GCA_976986655	GCA_976986645
Assembly identifier	drRubCham1.1.h1	drRubCham1.1.h2
Span (Mb)	1,198	1,161
Number of contigs	1,840	874
Contig N50 length (Mb)	29.4	33.1
Longest contig (Mb)	51.3	51.5
Number of gaps	25	24
Number of scaffolds	1,815	850
Scaffold N50 length (Mb)	38.4	40.0
Longest scaffold (Mb)	51.4	51.5
Consensus quality (QV) compared to Hi-C (compared to HiFi)	45.9 (58.5)	47.0 (60.3)
Both assemblies	46.4 (59.3)	
*k*-mer completeness (%) compared to Hi-C (compared to HiFi)	96.0 (82.7)	94.0 (82.6)
Both assemblies	96.5 (98.6)	
Percentage of assembly mapped to chromosomes	93.6%	96.6%
Sex chromosomes	None	
Organelles^a^	MT1/MT2, PL (plastid)	
**Genome annotation metrics**		
Number of protein-coding genes	74,132	70,692
Number of protein-coding genes with functional domain^b^	72,128	68,578
Number of protein-coding genes with gene names	54,204	50,835
BUSCO^c^	C:99.2%[S:0.7%,D:98.5%],F:0.4%,M:0.4%,n:2326,E:1.0%	C:99.2%[S:1.0%,D:98.2%],F:0.3%,M:0.4%,n:2326,E:0.8%

a Both MT1 and MT2 are partial representations of the full *R. chamaemorus* mitochondria.

b Number of genes annotated with a functional domain as found by InterProScan.

c BUSCO scores based on the eudicots BUSCO set using v5.4.7.

C = complete [S = single copy, D = duplicated], F = fragmented, M = missing, *n* = number of orthologues in comparison.

When compared to a k-mer database of the Hi-C reads, hap1 had a k-mer completeness of 96.0%, hap2 of 94.0%, and combined they have a completeness of 96.5%. Further, hap1 had an assembly consensus quality value (QV) of 45.9 and hap2 of 47.0, where a QV of 40 corresponds to 1 error every 10,000 bp, or 99.99% accuracy compared to a k-mer database of the Hi-C reads (QV 58.5 and 60.3, respectively, compared to a k-mer database of the HiFi reads) (Table 1). When comparing the 2 pseudo-haplotypes using minimap2, there are 1,775,487 single nucleotide polymorthism (SNP) differences (2.098% of the aligned sequence), 149,334 deletions in hap2 compared to hap1, ranging from 1 bp to more than 1,000 bp, and 149,763 insertions from 1 bp to more than 1,000 bp in size (Supplementary Table 2). A total of 74,132 and 70,692 protein-coding genes were annotated in hap1 and hap2, respectively (Table 1).

The Hi-C contact maps show clear separation of chromosomes into homologous sets (Supplementary Fig. 1), and show no significant signs of contamination (Supplementary Fig. 2). When sorted by sequence similarity inferred from Hi-C contact patterns rather than by chromosome size, each pseudo-haplotype reveals 7 distinct groups of 4. Repeat masking with RED (Girgis 2015) identified a high proportion of repetitive content, typical for plant genomes: 60.1% in hap1 and 59.6% in hap2.

**Fig. 3:**
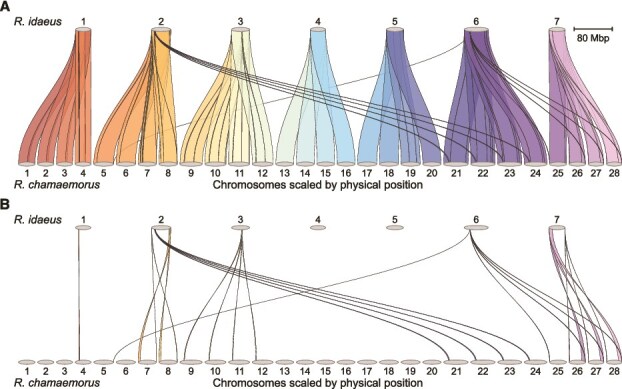
Synteny between *R. chamaemorus* (cloudberry) and *R. idaeus* (raspberry). Both panels are riparian plots generated using GENESPACE. Each *R. idaeus* chromosome aligns with 4 distinct *R. chamaemorus* hap1 chromosomes, consistent with an octoploid origin. Colors follow a continuous gradient for visual clarity. A) Full syntenic alignment. B) Alignment plot with conserved collinear blocks manually removed in Inkscape to highlight structural differences (e.g. inversions, translocations).

Using the phased assembly, we assessed genome-wide synteny between *R. chamaemorus* hap1 and *R. idaeus* (raspberry)*—*a closely related diploid species with a high-quality chromosome resolved assembly (Martinussen et al. 2013; Price et al. 2023). The synteny analysis revealed a clear 4:1 chromosome correspondence with limited large-scale rearrangement, supporting an octoploid origin within *Rubus* (Fig. 3). While most *R. idaeus* chromosomes align directly with 4 *R. chamaemorus* homeologs, several minor deviations indicate historical structural rearrangements. Chromosome 2 in *R. idaeus* shows near-complete synteny with *R. chamaemorus* chromosomes 5 to 8, but also a small translocation to chromosomes 21 to 24 (Fig. 3B). A similar pattern is seen for *R. idaeus* chromosome 6, which aligns primarily with chromosomes 21 to 24 but also shows a small translocation to chromosomes 25 to 28, and a unique translocation from *R. idaeus* chromosome 6 to *R. chamaemorus* chromosome 5. *R. idaeus* chromosome 3 has a smaller central region syntenic across all 4 homologs of *R. chamaemorus* (chromosomes 9 to 12). There is also evidence of large inversions. A large central inversion is the simplest explanation for the synteny break between *R. idaeus* chromosome 2 and chromosomes 7 to 8, and a similar inversion pattern is observed between chromosome 7 and chromosomes 26 to 28 (Fig. 3B). *R. chamaemorus* chromosome 25 is structurally distinct, lacking this inversion.

Hap1 had 99.2% and hap2 99.2% complete BUSCO genes using the eudicots lineage set. Each pseudo-haplotype most commonly has 4 BUSCO copies, consistent with the known octoploid status of *R. chamaemorus* (Table 2). The next most frequent categories are 3 copies, likely reflecting gene loss (fractionation), and >4 copies, which may result from recent small-scale duplications or assembly noise. The presence of 2-copy BUSCOs (7.39%) in diploid *R. idaeus* suggests that some genes in the eudicot BUSCO set may not be strictly single-copy within *Rubus*.

**Table 2:** BUSCO duplicates in *R. chamaemorus* compared to *R. idaeus.*

# BUSCO copies	*Rubus chamaemorus* hap 1	*Rubus chamaemorus* hap 2	*Rubus idaeus*
0 (missing)	10 (0.43)	10 (0.43)	32 (1.38)
1	26 (1.12)	32 (1.38)	2,143 (90.76)
2	66 (2.84)	58 (2.49)	172 (7.39)
3	467 (20.08)	461 (19.82)	10 (0.43)
4	1,648 (70.85)	1,659 (71.32)	1 (0.04)
>4	109 (4.69)	106 (4.56)	0
Total	2,326	2,326	2,326

**Fig. 4:**
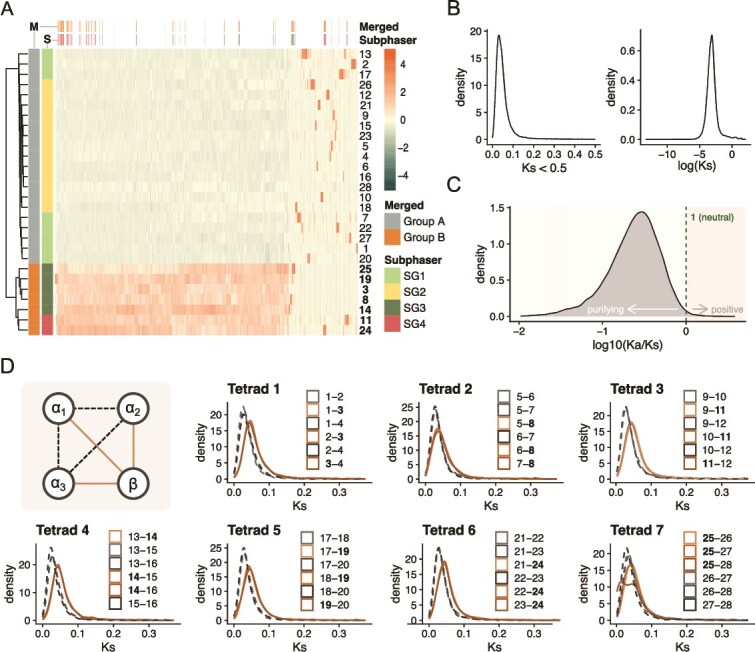
Subgenome structure inferred from k-mer and Ks analyses. A) Unsupervised hierarchical clustering of k-mer profiles via SubPhaser. Top bar: k-mer subgenome specificity. Side bar: Chromosome set assignments. “Merged” labels reflect our inferred α/β sets based on a stricter interpretation of the clustering. Heatmap shows Z-scored relative abundance of 10,000 randomly sampled k-mers. B) Ks distribution of segmental duplicates (as defined by doubletrouble) across all chromosomes. Left: Linear scale; right: log10-transformed Ks. C) Ka/Ks distribution (filtered for Ks > 0.05, ka/Ks > 0.01; artifact removal), showing most duplicates are under strong purifying selection. D) Ks distributions of segmental duplicates within each of the 7 syntenic pseudo-chromosome tetrads. The leftmost panel shows pairwise comparisons among the 4 homeologs in each tetrad. Solid (orange) lines indicate comparisons between each α-homeolog and the corresponding β-homeolog. Stippled (gray) lines indicate comparisons among the 3 α-homeologs.

To explore the evolutionary history of polyploidization events in *R. chamaemorus*, we first used SubPhaser (Jia et al. 2022) to identify subgenome-specific k-mers and cluster homoeologous chromosomes into putative subgenomes (Fig. 4A). The analysis identified 1 distinct set of 7 chromosomes, hereafter termed the β-set (red, Fig. 4A), but was unable to separate the remaining 21 chromosomes into putative subgenomes; these were therefore collectively grouped as the α-set (gray, Fig. 4A).

We then estimated Ks distributions using doubletrouble (Almeida-Silva and Van de Peer 2025). The overall distribution showed a single, narrow peak with only a slight shoulder on the right (Fig. 4B). Ka/Ks ratios indicate strong purifying selection across most gene pairs, consistent with functional constraint and subgenome stability (Fig. 4C). Stratifying the analysis by tetrad (7 sets of 4 syntenic chromosomes), 6 of the 7 showed a consistent pattern: 1 chromosome had higher pairwise Ks values relative to the other 3 (Fig. 4D, Supplementary Table 3). These chromosomes correspond to the β-set identified in the k-mer analysis. The seventh tetrad displayed a more complex pattern. Pairwise Ks comparisons between chromosomes 25 (β) and 28 (α) show a bimodal distribution (Supplementary Fig. 3), with 1 peak overlapping the typical α–β divergence and a second peakat lower Ks values than those observed among α–α comparisons in other tetrads.

**Figure 5:**
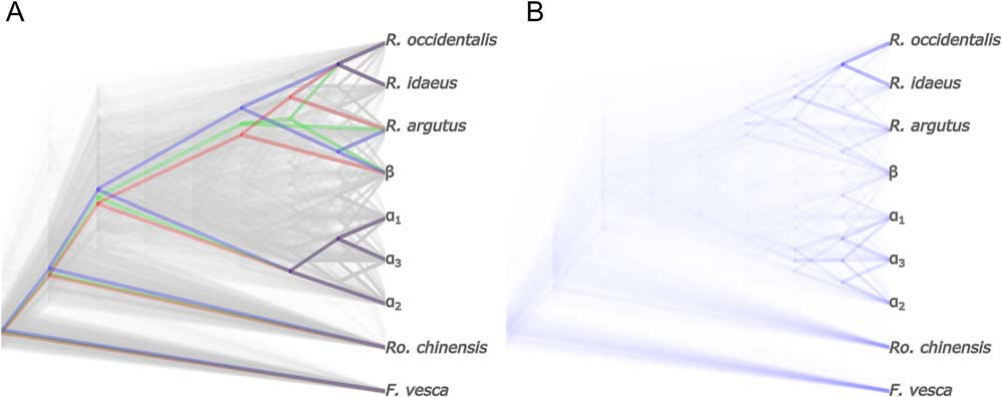
Gene tree structure across all retained loci. All retained gene trees (*n* = 2,674) were combined after relabeling *R. chamaemorus* α homeologous chromosomes (α1 to α3), based on their relative genetic similarity to the k-mer–distinguishable β chromosome (see Materials and methods). Trees were rooted using *F. vesca* (wild strawberry) as an outgroup. A) the full set of individual gene trees. The 3 most frequent topologies are highlighted (first: blue, second: red, third: green), while less frequent alternative topologies are shown in gray with lower opacity. B) DensiTree consensus visualization showing only consensus trees. Branch density reflects the frequency with which the same topological relationships recur across the full gene tree set.

To test whether the unresolved α set contains phylogenetic structure, we examined genome-wide gene-tree topology patterns among homoeologs. Throughout, α1 to α3 denote α homeologs ordered by sequence similarity to β (Methods). Gene trees show extensive topological conflict within *Rubus* (Fig. 5A; Supplementary Fig. 4C). No single dominant topology is apparent when all individual gene trees are visualized, consistent with the high proportion of unique gene tree topologies (74%). Despite this overall conflict, some relationships recur. In all 3 most common topologies (blue, red, green), *Rubus occidentalis* and *R. idaeus* consistently form a sister pair. By contrast, the placement of *Rubus argutus* is unstable and varies with the placement of the β homeolog.

Structurally, tetrad 7 uniquely shows terminal inversions in the β chromosome relative to all 3 α homeologs, whereas tetrad 2 shows similar end inversions relative to only 2 of the 3 α chromosomes; by contrast, tetrad 1 shows only a single internal inversion (Supplementary Fig. 4A). Concordantly, only tetrads 2 and 7 show β linking with α in the most frequent gene-tree topology (Supplementary Fig. 4C).

In the 3 most frequent topologies, using sequence similarity to β to name α-subsets, the homeologs α1 and α3 form a monophyletic group, and α2 recovered as sister to this group. However, together these topologies account for only ~ 4% (108) of the total tree set, while a large proportion of trees are singletons (~34%, 916), indicating substantial topological heterogeneity. As a result, when all trees are shown in the consensus visualization (Fig. 5B), no stable or consistently recurring relationship among the α homeologs is recovered, and they instead form a diffuse cluster reflecting substantial topological variation.

**Figure 6:**
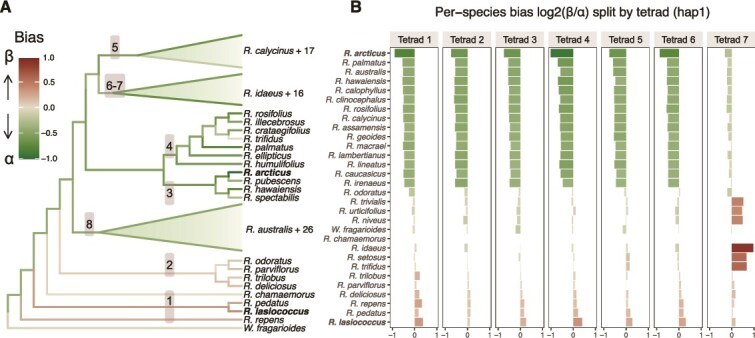
High-confidence read mappings (MAPQ ≥ 30) from Carter et al. (2019) depicting bias of *Rubus* species mapped to a reduced α and β set of *R. chamaemorus*. Reads from each species are mapped to *R. chamaemorus* haplotype 1 (hap1). Log₂ of the ratio of high-confidence reads mapped to the β-set over the α-set, with bias shifted so that *R. chamaemorus* is at 0. A value of 1 indicates a 2-fold mapping bias toward the β-set. Chromosomes from the α-set: 1, 5, 9, 13, 17, 21, 26; from the β-set: 3, 8, 11, 14, 19, 24, 25. A) *R. chamaemorus* centered bias (median across tetrads) projected onto the ASTRAL-II exon all-taxa tree; the tree is collapsed to show only relevant lineages (numbers denote groups defined by Carter et al. (2019). The most α-biased and β-biased species are marked in bold. B) Bias stratified by chromosome pair (tetrad). Species are ordered from most α-biased (most negative) to most β-biased (most positive). Only the 15 most α-biased and the 15 least α-biased species are shown.

Given the presence of a distinct β set, we assess whether reads from various *Rubus* taxa map preferentially to α or β*. R. pedatus* and *R. lasiococcus* have previously been proposed as progenitor species of *R. chamaemorus* (Michael 2006; Carter et al. 2019), while *R. arcticus* has been found to share a GBSSI-1γ copy with *R. chamaemorus *(**Michael 2006*)*. Although genome assemblies are not available for these species, limited sequencing data is available (through Carter et al. (2019) and Kates et al. (2024); See Materials and methods). To minimize mapping ambiguity among the indistinguishable α subsets, we retained only the longest α representative per tetrad and quantified high-confidence alignments across α and β. Onto each phylogenetic tree, we painted log₂ coverage bias (Fig. 6; full species lists and complete trees in Supplementary Figs 5 to 8).

In the Carter dataset, we observe the strongest β bias within the *R. pedatus–R. lasiococcus* clade, with weak β bias or no bias in other early-diverging *Rubus* lineages (Fig. 6A; group 2). The main *Rubus* clade is predominantly α-biased in Carter, with a few neutral species nested within otherwise α-biased subclades (Supplementary Figs 5 to 6). When the signal is examined by tetrad, tetrad 7 (chr25–28; chr25 vs. 26) behaves idiosyncratically: *R. idaeus* exhibits an anomalously strong β bias (Fig. 6B), and several otherwise weakly biased species show the same pattern specifically for this tetrad. Neither *R. idaeus* nor these other species show comparable β bias in any other tetrads.

In the Kates dataset, overall β bias is stronger than in Carter, shifting groups 5 to 8 to be neutral/slightly β-biased, with *R. pedatus* showing the strongest β bias and *R. arcticus* and associated clades (3 to 4) retaining their α-biased status (Supplementary Figs 7 and 8). While the overall bias in Kates is either neutral or β-biased, tetrad 6 stands out with its strong and consistent α-bias. Unlike in Carter, there are spikes of β-bias in tetrads 4 and 5, as well as tetrad 7, which might be a result of fewer and less informative loci for *Rubus* in this dataset.

### Subgenome evolution scenarios

**Figure 7:**
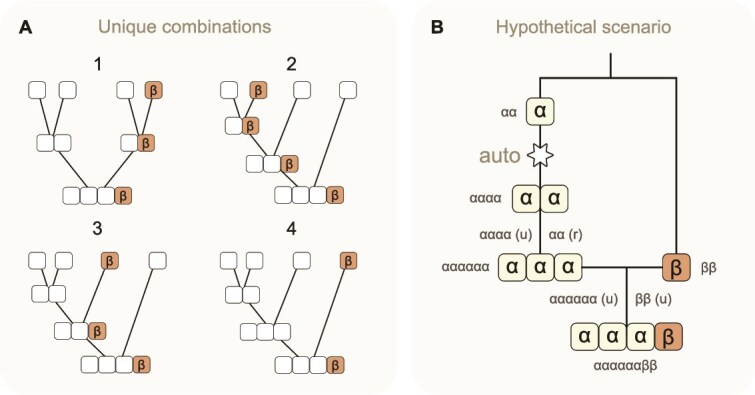
A hypothetical scenario for subgenome origin and polyploidization. A) When only 1 of 4 subgenomes are distinguishable, 15 possible octoploid topologies collapse into 4 equivalence classes; 1 where the intermediates are tetraploids (1), and 3 where a hexaploid is an intermediate (2 to 4). B) Hypothetical scenario based on (A-4): Autotetraploidization of α, followed by fusion of reduced (r) and unreduced (u) α gametes to form an autohexaploid. Subsequent fusion of unreduced α and β gametes yields an auto-allo octoploid, in which no α subset shares a distinct evolutionary history with β.

Together, our results (see Figs 3 to 6) constrain the set of plausible polyploidization pathways leading to the observed octoploid. Across k-mer composition, Ks profiles, and genome-wide gene-tree topology, 1 set of 7 chromosomes is consistently distinct (β), whereas the remaining 21 (3 × 7) chromosomes form an α set with no clear resolvable internal subdivision. Under this constraint, many detailed histories collapse into a small number of equivalence classes (Fig. 7A), differing mainly in whether a tetraploid or hexaploid intermediate is invoked and in the order of β incorporation. We present 1 hypothetical scenario (Fig. 7B) to illustrate how the α-set could arise lacking any internal structure prior to the incorporation of a distinct β lineage. Supplementary Fig. 9 provides additional representative (non-exhaustive) alternatives consistent with the same constraints.

## Discussion

Polyploidy is pervasive in *Rubus*, with 60% to 70% of species possessing chromosome numbers 4n or higher (Thompson 1997; Hummer et al. 2016; Chen et al. 2020). Comparative synteny between *R. chamaemorus* and *R. idaeus* reveals a highly collinear subgenome structure, with 4 homologs corresponding to each of the 7 basal chromosomes typical of *Rubus*.

K-mer clustering, Ks analyses, and gene-tree topology indicate that 3 of the 4 chromosome sets are closely related and form an unresolved α-set, while the fourth is more divergent (β) (Figs 4 and 5). Gene trees inferred by OrthoFinder show extensive topological discordance, with a high proportion of unique topologies (Fig. 5). This lack of stable phylogenetic structure argues against the presence of 3 independently segregating α subgenomes. The lack of reproducible structure within the α set is most consistent with an autohexaploid or near-autohexaploid origin, in which closely related chromosome copies arise wholly or partly through autopolyploidization, rather than representing 3 independently segregating α subgenomes (Fig. 7B). This interpretation does not imply a single evolutionary pathway, as multiple distinct and potentially overlapping routes could give rise to an unresolved α set consistent with an autohexaploid state (Supplementary Fig. 9A). These inferences are necessarily limited by the small number of chromosome-scale *Rubus* assemblies currently available.

When assessing α–β mapping bias, both short-read datasets recover a consistent pattern, with β affinity increased in the *R. pedatus–R. lasiococcus* lineage and α affinity within the main *Rubus* clade, especially *R. arcticus* (Fig. 6). Although *R. pedatus* and *R. lasiococcus* have been proposed as progenitors of *R. chamaemorus* (Michael 2006), our results suggest they may account for only the β-set. By contrast, α affinity is strongest in lineages near *R. arcticus*, although the available data likely do not allow us to identify a specific α donor or distinguish between direct ancestry and related lineages. Coarse Ks-based estimates place the α–β divergence broadly in the Miocene (~6 to 14 Ma).

Tetrad 7 (chromosomes 25 to 28) consistently departs from the otherwise clear α–β pattern across analyses. Only the k-mer analysis cleanly assigns chromosome 25 clearly to β and chromosomes 26 to 28 to α. Pairwise Ks within the tetrad do not follow the typical α–β divergence pattern, with chr. 25 vs. 28 showing a bimodal Ks distribution (Supplementary Fig. 3). β in tetrad 7 (chr. 25) lacks the inversion present in all 3 α homeologs and instead shares gene order with red raspberry (Fig. 3), a pattern consistently supported by Hi-C and pairwise similarity analyses in both haplotypes (Supplementary Fig. 4A). Although the most frequent gene-tree topologies are rare overall, tetrads 7 (and 2) uniquely show the most frequent topology grouping β to α (Supplementary Fig. 4C), suggesting a localized deviation from the genome-wide pattern. Mapping-bias analysis further highlights this anomaly, with several species, most notably *R. idaeus*, showing a stark shift toward β only in this tetrad (Fig. 6B). Although assembly artifacts cannot be ruled out, these consistent irregularities across methods point to a complex evolutionary history for tetrad 7.

Distinguishing among these alternative histories will require additional data at 2 levels: population-level sampling within *R. chamaemorus* to evaluate inheritance patterns, and chromosome-scale genome assemblies from closely related *Rubus* species to resolve subgenome ancestry and the sequence of polyploidization events, particularly in regions showing localized departures from the genome-wide α–β pattern.

Beyond resolving genome structure and evolutionary history, the haplotype-resolved, chromosome-scale assemblies presented here provide a genomic framework for downstream trait-focused analyses in *R. chamaemorus*. One relevant application is primocane fruiting, a flowering-time trait present in several *Rubus* species (Jennings 2018), for which comparative analyses have identified candidate flowering-related loci in blackberry (Brůna et al. 2023a). In *R. chamaemorus*, the assemblies allow flowering-related homologs to be examined in genomic context and support the development of targeted assays for future genetic and functional studies.

## Materials and methods

### Sample acquisition and DNA extraction

The sample was derived from an adult male plant collected in a forested area of Ås, Norway (59.670983, 10.789649) on the 29 May 2022 (Fig. 1A). Sampling of this plant material does not require a specific permit under Section 15 of the Norwegian Nature Diversity Act, provided that it does not threaten the viability of the population and is not otherwise restricted.

DNA isolation for PacBio long read sequencing was performed using Circulomics Nanobind CBB BIG DNA kit and protocol according to manufacturer’s recommendations, including treatment with EtOH removal buffer (Circulomics, now PacBio company). Quality check of amount, purity, and integrity of isolated DNA was performed using Qubit BR DNA quantification assay kit (Thermo Fisher), Nanodrop (Thermo Fisher), and Fragment Analyzer (DNA HS 50 kb large fragment kit, Agilent Tech.).

### Library preparation and sequencing for de novo assembly

Before PacBio HiFi library preparation, DNA was purified an additional time using AMPure PB beads (1:1 ratio). Purified high-molecular-weight (HMW) DNA was sheared into an average fragment size of 15 to 20 kbp large fragments using the Megaruptor3 (Diagenode). A HiFi library was prepared following the PacBio protocol for HiFi library preparation using the SMRTbell® Prep Kit 3.0. The final HiFi library was size-selected with a 10 kbp cut-off using a BluePippin (Sage Sciences) and sequencing was performed by the Norwegian Sequencing Centre on a PacBio Sequel IIe instrument (Pacific Biosciences Inc). The library was sequenced on two 8 M SMRT cells using a Sequel II Binding kit 3.2 and Sequencing chemistry v2.0.

A Hi-C library was prepared using the Arima High Coverage HiC kit (Arima Genomics), following the manufacturer’s recommendations and starting with 1,050 mg leaf tissue. Chromatin was crosslinked prior to nuclei isolation as written in the user guide for plant tissues (Doc A160163 v01). Final library quality was quantified using a Kapa Library quantification kit for Illumina (Roche Inc.). The library was sequenced with other libraries on an Illumina NovaSeq 6000 (Illumina Inc) with 2 × 150 bp paired-end mode at the Norwegian Sequencing Centre (https://www.sequencing.uio.no).

### Genome assembly and curation, annotation, and evaluation

A full list of relevant software tools and versions is presented in Supplementary Table 1. We assembled the species using a pre-release of the EBP-Nor genome assembly pipeline (https://github.com/ebp-nor/GenomeAssembly).

### Sequencing data processing and k-mer analyses

KMC (Kokot et al. 2017) was used to count k-mers of size 32 in the PacBio HiFi reads, excluding k-mers occurring more than 10,000 times. GenomeScope (Ranallo-Benavidez et al. 2020) was run as part of the pipeline on the k-mer histogram output from KMC and was included in the methods for completeness, but as it does not support ploidy above 6, we do not report its results. Ploidy level was investigated using Smudgeplot (Ranallo-Benavidez et al. 2020). HiFiAdapterFilt (Sim et al. 2022) was applied on the HiFi reads to remove possible remnant PacBio adapter sequences. The filtered HiFi reads were assembled using Hifiasm (Cheng et al. 2021) with Hi-C integration resulting in a pair of haplotype-resolved assemblies, pseudo-haplotype 1 (hap1) and pseudo-haplotype 2 (hap2). Unique k-mers in each assembly/pseudo-haplotype were identified using meryl (Rhie et al. 2020) and used to create 2 sets of Hi-C reads, 1 without any k-mers occurring uniquely in hap1 and the other without k-mers occurring uniquely in hap2.

### Haplotype-resolved genome assembly

K-mer filtered Hi-C reads were aligned to each scaffolded assembly using BWA-MEM (Li 2013) with -5SPM options. The alignments were sorted based on name using samtools (Li et al. 2009) before applying samtools fixmate to remove unmapped reads and secondary alignments and to add mate score, and samtools markdup to remove duplicates. The resulting BAM files were used to scaffold the 2 assemblies using YaHS (Zhou et al. 2023) with default options. FCS-GX (Astashyn et al. 2024) was used to search for putative contamination, and contaminated sequences were removed. The mitochondrion was searched for in contigs and reads using Oatk (Zhou et al. 2025).

### Manual curation and chromosome assignment

The assemblies were manually curated using PretextView and Rapid Curation 2.0. Chromosomes were identified by mapping to *R. idaeus* JASCWY01 (Price et al. 2023) and *Fragaria vesca* v4 (Edger et al. 2018) in addition to inspecting the Hi-C contact map in PretextView.

### Genome annotation

We annotated the genome assemblies using a pre-release version of the EBP-Nor genome annotation pipeline (https://github.com/ebp-nor/GenomeAnnotation). First, AGAT (https://zenodo.org/record/7255559) agat_sp_keep_longest_isoform.pl and agat_sp_extract_sequences.pl were used on the *Arabidopsis thaliana* (TAIR10.1, GCF_000001735.4) genome assembly and annotation to generate 1 protein (the longest isoform) per gene. Miniprot (Li 2023) was used to align the proteins to the curated assemblies. UniProtKB/Swiss-Prot (UniProt Consortium 2023) release 2023_02 in addition to the Viridiplantae part of OrthoDB v11 (Kuznetsov et al. 2023) were also aligned separately to the assemblies. RED (Girgis 2015) was run via redmask (https://github.com/nextgenusfs/redmask) on the assemblies to mask repetitive areas. GALBA (Brůna et al. 2023b; Stanke et al. 2006; Buchfink et al. 2015; Hoff & Stanke 2019; Li 2023) was run with the *Arabidopsis* proteins using the miniprot mode on the masked assemblies.

The funannotate-runEVM.py script from Funannotate was used to run EvidenceModeler (Haas et al. 2008) on the alignments of TAIR10.1 proteins, UniProtKB/Swiss-Prot proteins, Viridiplantae proteins, and the predicted genes from GALBA. The resulting predicted proteins were compared to the protein repeats that Funannotate distributes using DIAMOND blastp, and the predicted genes were filtered based on this comparison using AGAT. The filtered proteins were compared to the UniProtKB/Swiss-Prot release 2023_02 using DIAMOND (Buchfink et al. 2015) blastp to find gene names and InterProScan (Jones et al. 2014) was used to discover functional domains. AGATs agat_sp_manage_functional_annotation.pl was used to attach the gene names and functional annotations to the predicted genes. EMBLmyGFF3 (Norling et al. 2018) was used to combine the FASTA files and GFF3 files into an EMBL format for submission to European Nucleotide Archive.

### Assembly quality assessment

All the evaluation tools have also been implemented in a pipeline, similar to assembly and annotation (https://github.com/ebp-nor/GenomeEvaluation). Merqury (Rhie et al. 2020) was used to assess the completeness and quality of the genome assemblies by comparing them to the k-mer content of both the Hi-C reads and PacBio HiFi reads. BUSCO (Manni et al. 2021) was used to assess the completeness of the genome assemblies by comparing against the expected gene content in the eudicots lineage. Gfastats (Formenti et al. 2022) was used to output different assembly statistics of the assemblies.

BlobToolKit and BlobTools2 (Laetsch and Blaxter 2017), in addition to blobtk, were used to visualize assembly statistics. To generate the Hi-C contact map image, the Hi-C reads were mapped to the assemblies using BWA-MEM (Li 2013) using the same approach as above. Finally, PretextMap was used to create a contact map, which was visualized using PretextSnapshot.

To characterize the differences between the 2 pseudo-haplotypes, we ran a genome alignment using minimap2 (Li 2017) on the homologous chromosomes. The resulting alignment was processed with the minimap2-included paftools.js, producing a report listing the number of insertions, SNPs, and indels between the 2 pseudo-haplotypes.

### Comparative genomics and evolutionary analyses

#### Synteny and orthology inference

To demonstrate the duplicated nature of *R. chamaemorus*, we analyzed synteny between hap1 and *R. idaeus* (red raspberry cv. “Autumn Bliss”) using the R package pipeline GENESPACE (Lovell et al. 2022). *Rubus idaeus* was chosen as reference due to its close phylogenetic relationship and availability of a high-quality chromosome-scale assembly (Martinussen et al. 2013; Price et al. 2023). To improve orthogroup inference, we included additional *Rubus*: *R. occidentalis* (v3.0—black raspberry) and *R. argutus* (“Hillquist” v1.0—blackberry), as well as more distantly related Rosaceae members: *Fragaria vesca* (v4.0.a1—wild strawberry) and *Rosa chinensis* (“Old Blush” v2.0—Chinese rose). Assemblies, protein sequences, and annotations were obtained from the Rosaceae database (https://www.rosaceae.org/). We selected the longest protein isoform as the representative for each gene, along with gene rank orders, to serve as input for GENESPACE. The pipeline was run with the default method, which uses OrthoFinder (Emms and Kelly 2019) to build orthogroups and MCScanX (Wang et al. 2012) to detect collinear regions. All other parameters were left at default. Synteny blocks between *R. chamaemorus* and *R. idaeus* were then visualized using the plot_riparian function of the GENESPACE package.

#### Subgenome inference and subgenome ancestry assessment

Subgenome structure in *R. chamaemorus* was assessed using SubPhaser v1.2.6 (Jia et al. 2022). The analysis was run independently on each haplotype, with default parameters and the 7 sets of 4 homologous chromosomes (tetrads) defined via a configuration file.

The doubletrouble R-package (Almeida-Silva and Van de Peer 2025) was used to calculate pairwise Ks values between segmental duplicates (i.e. duplicated genes in conserved syntenic blocks) in the hap1 assembly. Since we could not reliably group chromosomes into 4 subgenomes based on shared k-mer signatures found by SubPhaser (Fig. 4A), pairwise Ks values were instead stratified by chromosome tetrads.

To estimate divergence times, Ks values were filtered to exclude values greater than 2 to minimize the effects of substitutional saturation. Divergence time (T) was calculated using the relationship T = Ks/(2 × μ), where μ is the synonymous substitution rate. We calculated estimates using the range of rates reported for herbaceous angiosperms by Kay et al. (2006) (1.72 × 10^−9^ to 8.34 × 10^−9^ substitutions/site/year).

To assess potential parental contributors to the *R. chamaemorus* subgenomes, we analyzed mapping bias from available *Rubus* species (plus outgroup *Waldsteinia fragarioides*) using 2 datasets: Carter et al. (2019) “Target Capture Sequencing Unravels *Rubus* Evolution” (PRJNA510412) and Kates et al. (2024) “Shifts in Evolutionary Lability Underlie Independent Gains and Losses of Root-Nodule Symbiosis in a Single Clade of Plants” (PRJNA1022323, PRJNA1022030, PRJNA1022147, PRJNA1022025, PRJNA1022141, PRJNA1022138, PRJNA1022032, PRJNA1022029, PRJNA1022027, PRJNA1022023, PRJNA1022015, PRJNA1021620, PRJNA1021608). To reduce ambiguity among similar α copies, we retained only the longest α chromosome per tetrad together with the complete β subgenome and aligned reads to this reduced α + β reference with Bowtie2. We kept high-confidence alignments (MAPQ ≥30) to limit mismapping across homeologous regions, quantified bias between α and β chromosome pairs as the log2 ratio of read densities (reads per chromosome length) for β vs. α [log2(β/α)], and centered each pair so that *R. chamaemorus* had a bias of 0. For each dataset, we projected median species estimates onto the accompanying phylogeny, rooting on *W. fragarioides*: for Carter et al. (2019), the ASTRAL-II exon all-taxa tree (Exon_alltaxa_AstralII.tre), and for Kates et al. (2024)  Supplementary Dataset 3 (41467_2024_48 036_MOESM6_ESM.txt).

#### Chromosome-scale similarity mapping and graph construction

The *R. chamaemorus* genome is highly repetitive and exhibits strong sequence similarity among homeologous chromosomes, complicating conventional whole-genome alignment. To quantify chromosome-scale sequence similarity while limiting repeat-driven signal inflation, we used MashMap (Kille et al. 2023) to estimate coarse-scale similarity across all chromosomes from both haplotypes. MashMap uses sketch-based k-mer mapping to summarize long-range sequence similarity while reducing the influence of high-copy k-mers. Chromosomes from hap 1 and hap 2 were compared in a square all-against-all framework using a permissive filtering strategy (filter_mode = none) to retain weaker homeologous mappings. Mapping was restricted to segments with a minimum nucleotide identity of 80% (--pi 80), self-mappings were excluded (−X), and k-mer filtering was relaxed (--kmerThreshold 0.01) to improve sensitivity among highly similar chromosomes. MashMap blocks were filtered to retain only long homologous segments (≥200 kb). For each chromosome pair, these segments were combined to estimate the total amount of homologous sequence and its average nucleotide identity. Similarity between chromosomes was then defined as homologous sequence coverage, weighted by sequence identity and normalized by chromosome length, and summarized as a single symmetric similarity score. Pairwise dotplots for each tetrad are shown in Supplementary Fig. 4A.

To visualize chromosome relationships, the symmetric similarity matrix was converted into a directed graph by retaining, for each chromosome, its top 3 most similar partners (K = 3). Nodes represent chromosomes, and directed edges indicate the strongest similarity relationships. Within each tetrad, 1 homeologous chromosome was designated as β based on SubPhaser k-mer–based inference, and the remaining 3 were labeled α1 to α3 by decreasing similarity to β. The resulting directed similarity graph and inferred α/β ranks are shown in Supplementary Fig. 4B.

#### DensiTree visualization

We analyzed OrthoFinder-defined orthogroups in which *R. chamaemorus* contributed 4 genes, and each other species contributed exactly 1 gene. Only gene trees in which the 4 *R. chamaemorus* genes corresponded to a single chromosome tetrad were retained, yielding 2674 gene trees. For each retained tree, *R. chamaemorus* tips were relabeled as β or α1 to α3 using the graph-derived ranking for the corresponding tetrad. Trees were rooted using *F. vesca*, branch lengths were replaced with topology-derived Grafen lengths, and tip order was standardized for visualization.

Relabeled trees were visualized in DensiTree2 both as a combined set across all tetrads and as separate per-tetrad sets (Supplementary Fig. 4C). Leaf order was optimized using DensiTree’s distance-based shuffle (“Closest Outside First”) to reduce visual clutter.

Parts of the text in Methods and Results are based on a template we use for all the species we publish in the EBP-Nor project.

## Supplementary Material

JOH_review_updated_supplementary_esag028

## Data Availability

All data are available in the European Nucleotide Archive (ENA). The umbrella project for EBP-Nor is BioProject PRJEB98354. Raw PacBio HiFi reads for the drRubCham1 sample (BioSample SAMEA120268085) are deposited under run accessions ERR15658288 and ERR15658311. Illumina Hi-C sequencing reads are available under run accession ERR15658302. The 2 assemblies are archived separately: pseudo-haplotype 1 under BioProject PRJEB98298 (assembly GCA_976986655) and pseudo-haplotype 2 under BioProject PRJEB98353 (assembly GCA_976986645). Genome assemblies and gene annotations are also available at **10.5281/zenodo.15773906**
