# Supplementary material for: The origin of the octoploid cloudberry (Rubus chamaemorus) genome is the result of multiple and complex polyploidization events
Source: J Hered. 2026 Mar 25;117(4):822–33. doi: 10.1093/jhered/esag028 (PMC13326439; doi:10.1093/jhered/esag028)
Supplement: JOH_review_updated_supplementary_esag028 [file joh_review_updated_supplementary_esag028.pdf]

Supplementary Material

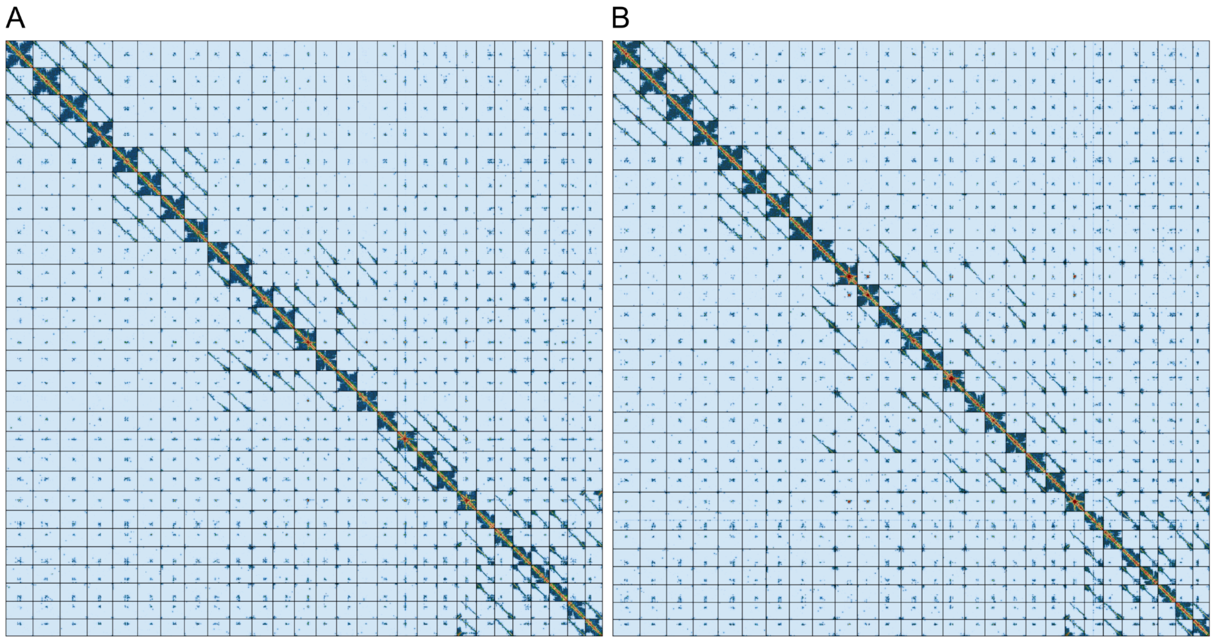

Supplementary Figure 1: Hi-C contact map of genome assemblies for *R. chamaemorus* hap1 and hap2. A) Hi-C contact map for hap1. B) Hi-C contact map for hap2. The assemblies are visualized using PreTextSnapshot. Chromosomes are shown in order of size from left to right and top to bottom.

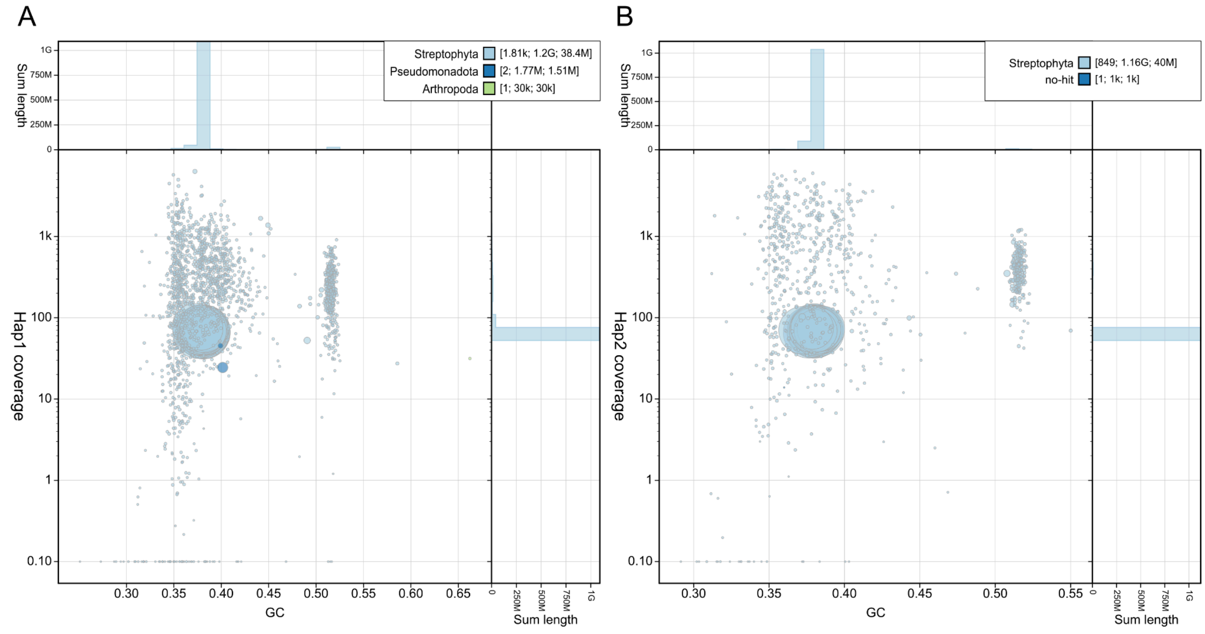

Supplementary Figure 2: BlobToolKit GC-coverage plots of genome assemblies of *R. chamaemorus* hap1 and hap2. A) GC-coverage plot for hap1. B) GC-coverage plot for hap2. The scaffolds are coloured by phylum. The size of the circles are in proportion to the length of the scaffolds. Histograms show the distribution of scaffold length sum along each axis.

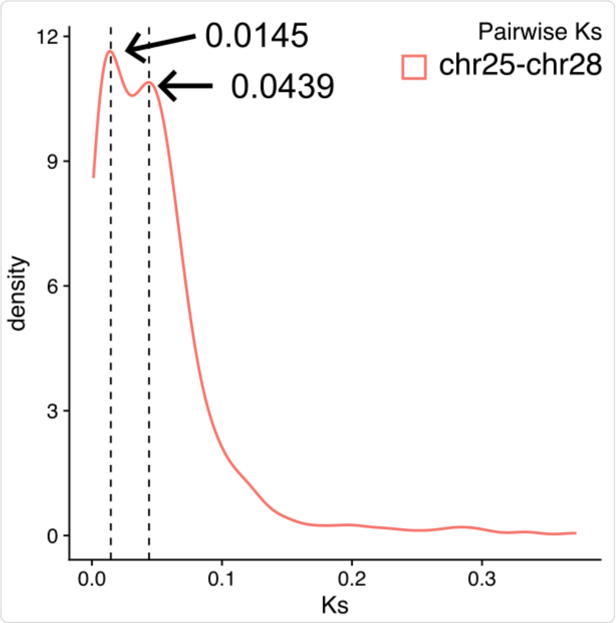

Supplementary Figure 3: Ks distribution between chromosomes 25 and 28 in Tetrad 7. Pairwise Ks distribution for chromosome pair 25–28 reveals two distinct peaks, unlike any other combination of chromosome pairs. Vertical dashed lines indicate the estimated Ks peak positions (~0.0145 and ~0.0439).

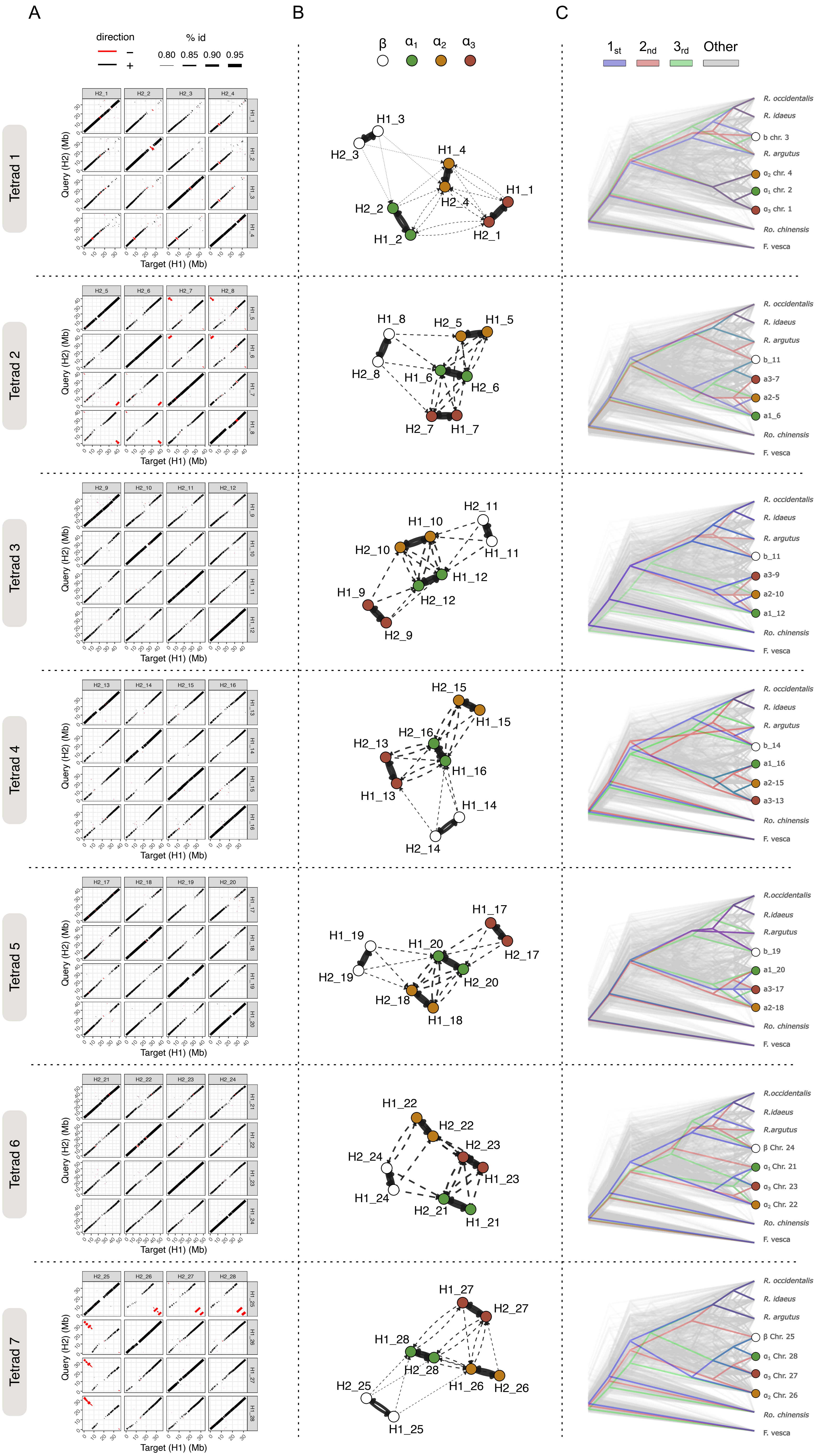
A
B
C
direction
–
+
% id
0.80 0.85 0.90 0.95
β α1 α2 α3
1st 2nd 3rd Other
Tetrad 1
Tetrad 2
Tetrad 3
Tetrad 4
Tetrad 5
Tetrad 6
Tetrad 7
Query (H2) (Mb)
Target (H1) (Mb)
H1_3
H2_3
H1_4
H2_4
H1_1
H2_1
H2_2
H1_2
H1_8
H2_8
H2_5
H1_5
H1_6
H2_6
H2_7
H1_7
H2_11
H1_11
H1_10
H2_10
H1_12
H2_12
H1_9
H2_9
H2_15
H1_15
H2_16
H1_16
H2_13
H1_13
H1_14
H2_14
H1_17
H2_17
H1_19
H2_19
H1_20
H2_20
H2_18
H1_18
H1_22
H2_22
H2_23
H1_23
H2_24
H1_24
H2_21
H1_21
H1_27
H2_27
H1_28
H2_28
H1_26
H2_26
H2_25
H1_25
R. occidentalis
R. idaeus
b chr. 3
R. argutus
α2 chr. 4
α1 chr. 2
α3 chr. 1
Ro. chinensis
F. vesca
b_11
a3-7
a2-5
a1_6
a3-9
a2-10
a1_12
b_14
a1_16
a2-15
a3-13
b_19
a1_20
a3-17
a2-18
β Chr. 24
α1 Chr. 21
α3 Chr. 23
α2 Chr. 22
β Chr. 25
α1 Chr. 28
α3 Chr. 27
α2 Chr. 26

Supplementary Figure 4. Chromosome-set labeling and gene tree signal across tetrads. A) Pairwise chromosome similarity dotplots between haplotypes. **B)** Directed chromosome similarity graph (K = 3), with nodes colored by β and α1 - α3 rank. **C)** DensiTree visualization of per-tetrad gene trees, with *R. chamaemorus* tips labeled by β/α rank and chromosome. The three most frequent topologies are highlighted (blue, red, green); all others are shown in gray. Apparent polytomies in panel C are a visualization effect. All gene trees are fully bifurcating; fixed tip positions in the DensiTree layout cause closely spaced splits to overlap.

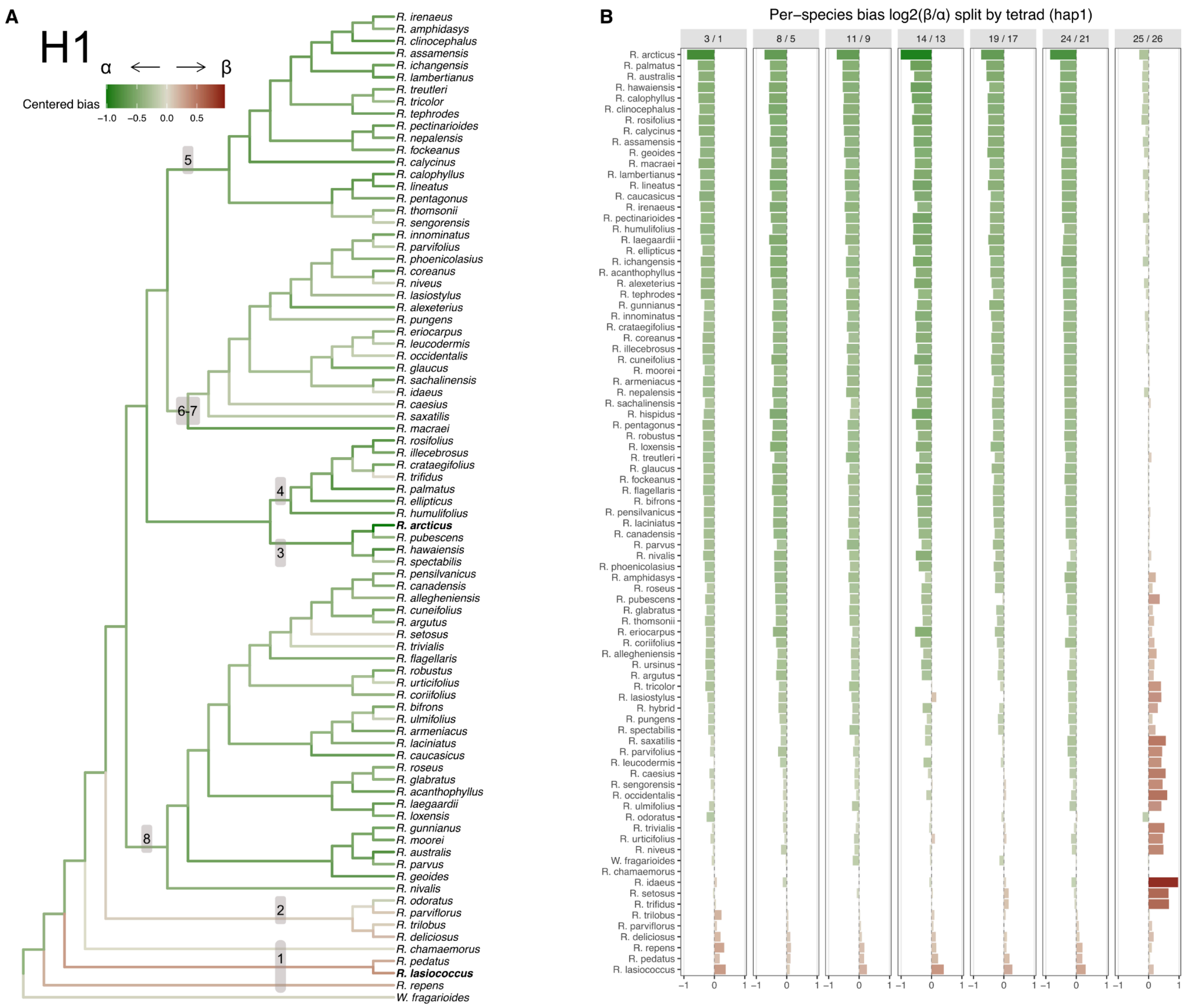

Supplementary Figure 5: High-confidence read mappings (MAPQ ≥ 30) from Carter et al. (2019) depicting bias of *Rubus* species mapped to hap1 of the reduced α and β subgenome set of *R. chamaemorus*. Reads from each species are mapped to *R. chamaemorus* haplotype one (hap1). Log_2 of the ratio of high-confidence reads mapped to subgenome β over subgenome α, with bias shifted so that *R. chamaemorus* is at 0. A value of 1 indicates a two-fold mapping bias toward subgenome β. Chromosomes from subgenome α: 1, 5, 9, 13, 17, 21, 26; from subgenome β: 3, 8, 11, 14, 19, 24, 25. **A)** Bias projected onto the ASTRAL-II exon all-taxa tree. (Numbers denote groups as defined by Carter et al. (2019, Fig. 2)). The α-biased and β-biased species are marked in bold. **B)** Bias stratified by chromosome pair (tetrad). For each tetrad, taxa are ordered by median bias from most α-biased (most negative) to most β-biased (most positive).

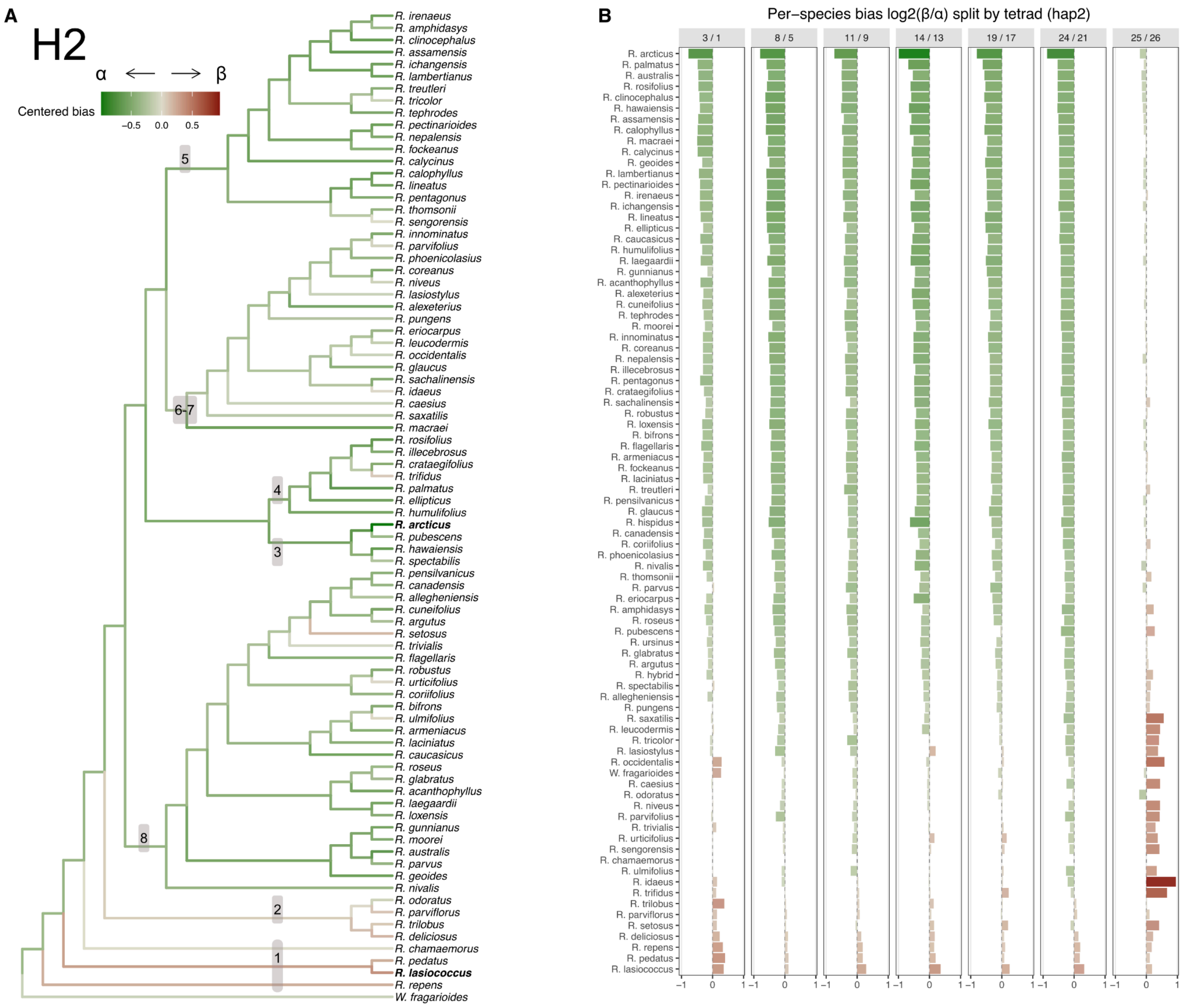

Supplementary Figure 6: High-confidence read mappings (MAPQ ≥ 30) from Carter et al. (2019) depicting bias of *Rubus species* mapped to hap2 of the reduced α and β subgenome set of *R. chamaemorus*. Reads from each species are mapped to *R. chamaemorus* haplotype two (hap2). $\log_2$ of the ratio of high-confidence reads mapped to subgenome β over subgenome α, with bias shifted so that *R. chamaemorus* is at 0. A value of 1 indicates a two-fold mapping bias toward subgenome β. Chromosomes from subgenome α: 1, 5, 9, 13, 17, 21, 26; from subgenome β: 3, 8, 11, 14, 19, 24, 25. **A)** Bias projected onto the ASTRAL-II exon all-taxa tree. (Numbers denote groups as defined by Carter et al. (2019, Fig. 2)). The α-biased and β-biased species are marked in bold. **B)** Bias stratified by chromosome pair (tetrad). For each tetrad, taxa are ordered by median bias from most α-biased (most negative) to most β-biased (most positive).

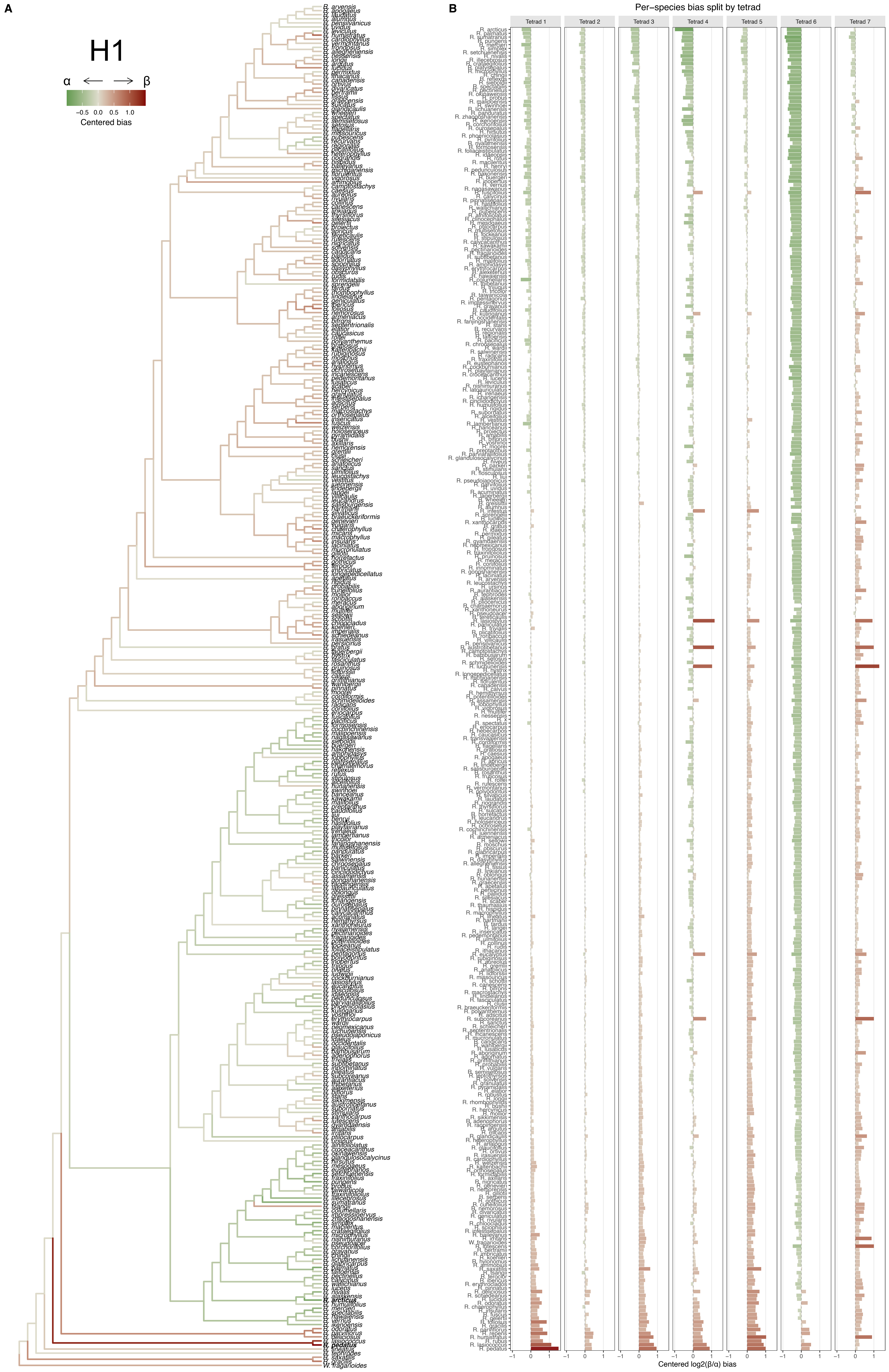
A
H1
α
β
−0.5 0.0 0.5 1.0
Centered bias
B
Per−species bias split by tetrad
Tetrad 1
Tetrad 2
Tetrad 3
Tetrad 4
Tetrad 5
Tetrad 6
Tetrad 7
Centered log2(β/α) bias

Supplementary Figure 7: High-confidence read mappings (MAPQ ≥ 30) from Kates et al. (2024) depicting bias of *Rubus* species mapped to hap1 of the reduced α and β subgenome set of *R. chamaemorus*. Reads from each species are mapped to *R. chamaemorus* haplotype one (hap1). Log_2 of the ratio of high-confidence reads mapped to subgenome β over subgenome α, with bias shifted so that *R. chamaemorus* is at 0. A value of 1 indicates a two-fold mapping bias toward subgenome β. Chromosomes from subgenome α: 1, 5, 9, 13, 17, 21, 26; from subgenome β: 3, 8, 11, 14, 19, 24, 25. **A)** Bias projected onto the Kates et al. 2024 supplementary dataset 3 phylogeny (41467_2024_48036_MOESM6_ESM.txt). The α-biased and β-biased species are marked in bold. **B)** Bias stratified by chromosome pair (tetrad). For each tetrad, taxa are ordered by median bias from most α-biased (most negative) to most β-biased (most positive).

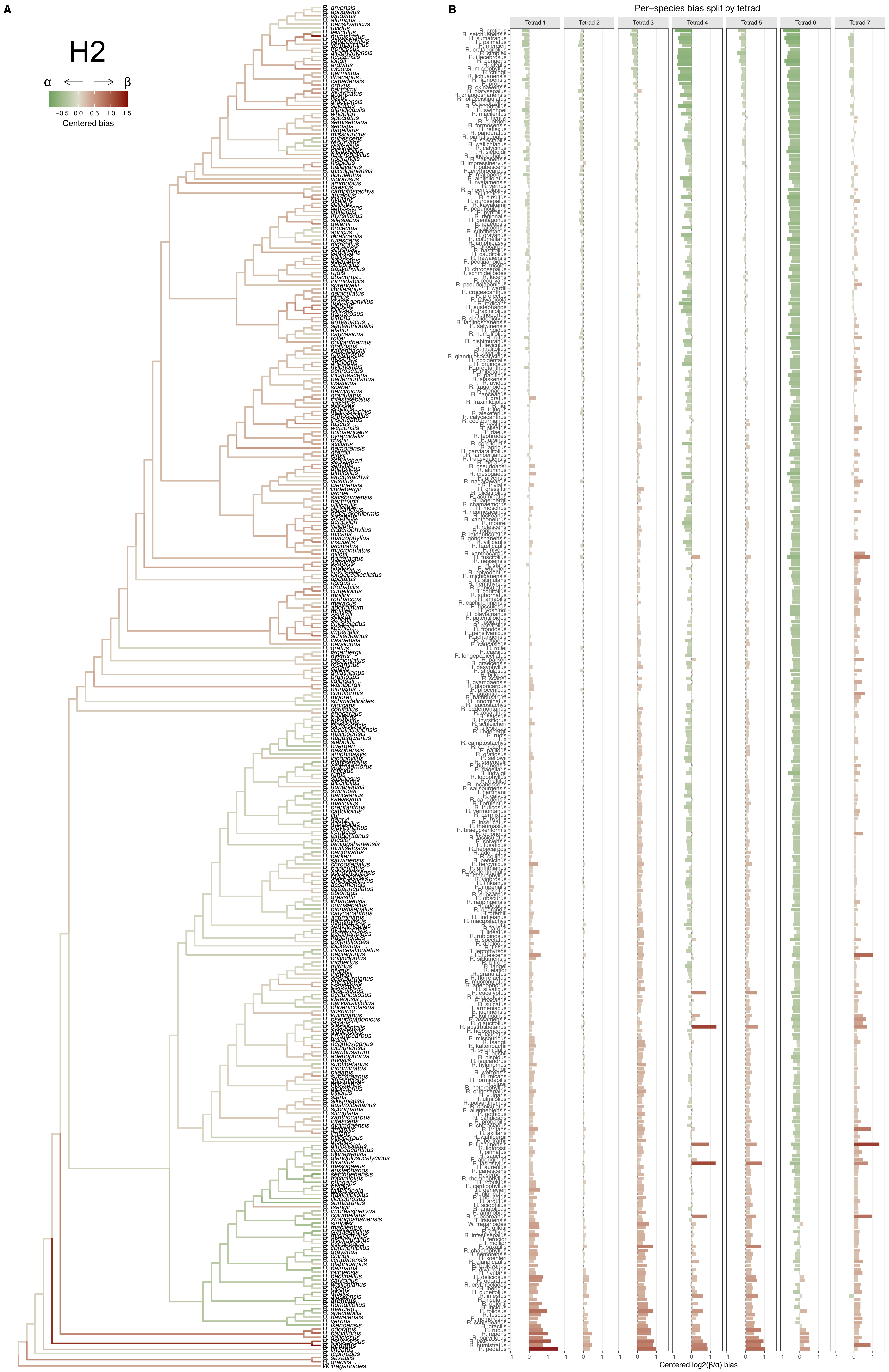
A
H2
α
β
−0.5 0.0 0.5 1.0 1.5
Centered bias
B
Per−species bias split by tetrad
Tetrad 1
Tetrad 2
Tetrad 3
Tetrad 4
Tetrad 5
Tetrad 6
Tetrad 7
Centered log2(β/α) bias

Supplementary Figure 8: High-confidence read mappings (MAPQ ≥ 30) from Kates et al. (2024) depicting bias of *Rubus* species mapped to hap2 of the reduced α and β subgenome set of *R. chamaemorus*. Reads from each species are mapped to *R. chamaemorus* haplotype two (hap2). Log_2 of the ratio of high-confidence reads mapped to subgenome β over subgenome α, with bias shifted so that *R. chamaemorus* is at 0. A value of 1 indicates a two-fold mapping bias toward subgenome β. Chromosomes from subgenome α: 1, 5, 9, 13, 17, 21, 26; from subgenome β: 3, 8, 11, 14, 19, 24, 25. **A)** Bias projected onto the Kates et al. 2024 supplementary dataset 3 phylogeny (41467_2024_48036_MOESM6_ESM.txt). The α-biased and β-biased species are marked in bold. **B)** Bias stratified by chromosome pair (tetrad). For each tetrad, taxa are ordered by median bias from most α-biased (most negative) to most β-biased (most positive).

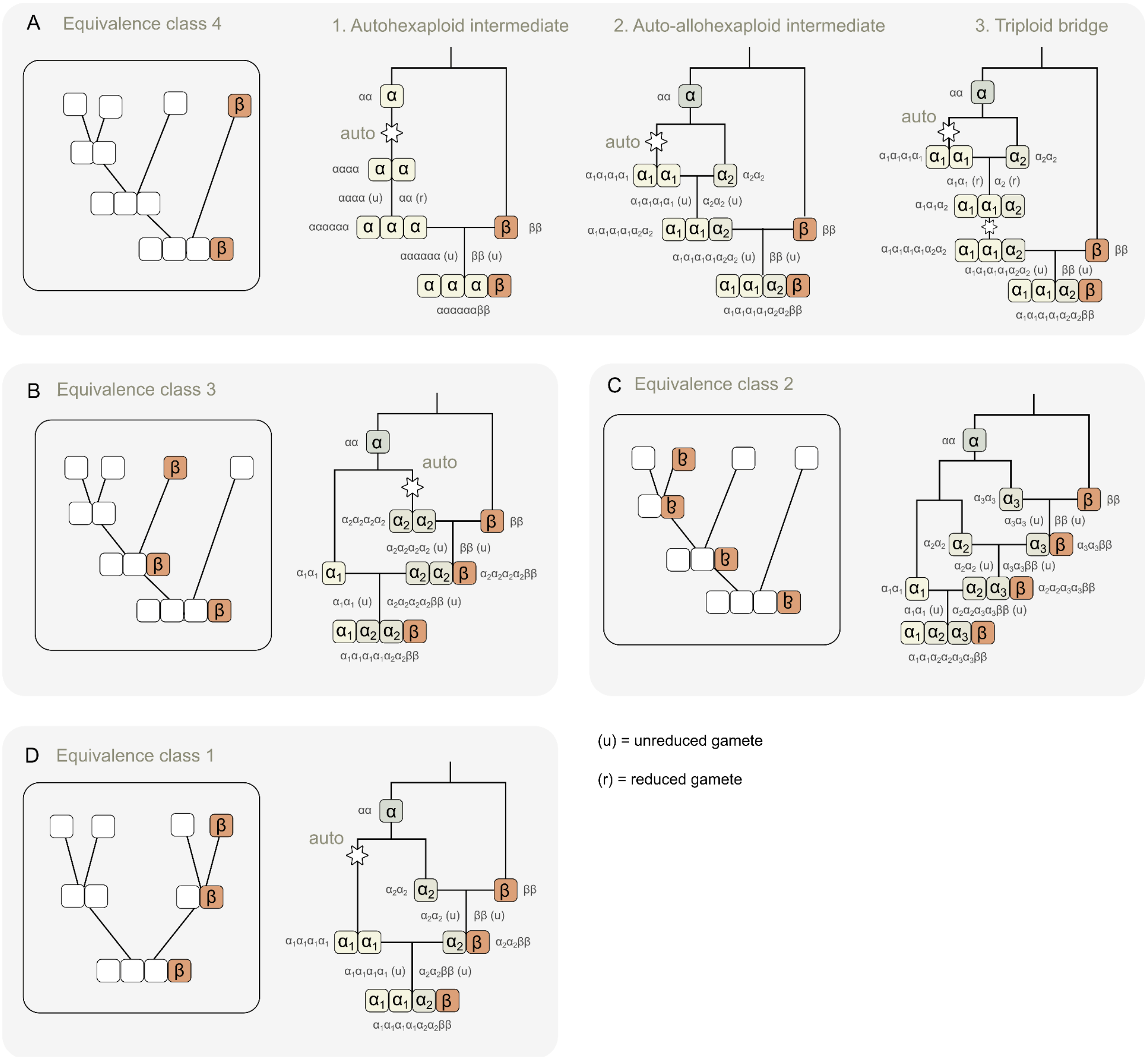

Supplementary Figure 9. Additional polyploidization scenarios consistent with the equivalence classes shown in Figure 7A. Panels A–D correspond to equivalence classes 4, 3, 2, and 1, respectively. Labels *r* and *u* shown adjacent to fusion points denote whether the contributing gamete in that event is reduced (*r*) or unreduced (*u*). Panel A illustrates multiple non-exhaustive scenarios within equivalence class 4, whereas panels B–D each show a single representative scenario for their respective classes.

Supplementary table 1. Software tools: versions and sources

Software tool	Version	Source
BlobToolKit	4.3.2	https://github.com/blobtoolkit/blobtoolkit
blobtk	0.5.8	https://github.com/blobtoolkit/blobtk
BUSCO	5.4.7	https://gitlab.com/ezlab/busco
Hifiasm	0.19.8	https://github.com/chhylp123/hifiasm
KMC	3.2.1	https://github.com/refresh-bio/KMC
GenomeScope	2.0	https://github.com/tbenavi1/genomescope2.0
HiFiAdapterFilt	2.0.1	https://github.com/sheinasim/HiFiAdapterFilt
PretextView	1.0.1	https://github.com/sanger-tol/PretextView
PretextMap	0.1.9	https://github.com/sanger-tol/PretextMap
PretextSnapshot	0.0.4	https://github.com/sanger-tol/PretextSnapshot
meryl	1.3.0	https://github.com/marbl/meryl
BWA-MEM	0.7.17	https://github.com/lh3/bwa
samtools	1.17	https://github.com/samtools/samtools
YaHS	1.2a.2	https://github.com/c-zhou/yahs
FCS-GX	0.4.0	https://github.com/ncbi/fcs
Merqury	1.3	https://github.com/marbl/merqury
AGAT	1.4.0	https://github.com/NBISweden/AGAT
Oatk	1.0	https://github.com/c-zhou/oatk
miniprot	0.13	https://github.com/lh3/miniprot

GALBA	1.0.9	https://github.com/Gaius-Augustus/GALBA
RED	2018.09.10	https://github.com/BioinformaticsToolsmith/Red
Funannotate	1.8.17	https://github.com/nextgenusfs/funannotate
EvidenceModeler	2.1.0	https://github.com/EVidenceModeler/EVidenceModeler
DIAMOND	2.1.8	https://github.com/bbuchfink/diamond
InterProScan	5.62-94.0	https://www.ebi.ac.uk/interpro/search/sequence/
EMBLmyGFF3	2.2	https://github.com/NBISweden/EMBLmyGFF3
Earl Grey	4.1.1	https://github.com/TobyBaril/EarlGrey
Rapid curation 2.0	964d17e997e00c69f25940cf96d3658bda631147	https://github.com/Nadolina/Rapid-curation-2.0,
GENESPACE	1.3.1	https://github.com/jtlovell/GENESPACE
Bowtie2	2.5.4	https://github.com/BenLangmead/bowtie2
MashMap	3.1.3	https://github.com/marbl/MashMap
DensiTree	3.1.0	https://www.cs.auckland.ac.nz/~remco/DensiTree/

Supplementary table 2. Alignment comparisons between assemblies of *Rubus chamaemorus* hap1 and hap2. Hap2 aligned to hap1.

Bases in alignment	731,366,624	
Substitutions (%)	1,775,487 (2.098%)	
Total # insertions	149,763	
Total # deletions	149,334	
	Insertions	Deletions
1bp	53,769	53,297
2bp	22,102	22,124
[3,50)	61,606	61,547
[50,1000)	7,110	7,023

>=1000	5,176	5,343

Supplementary table 3: Median Ks distances between tetrads of syntenic chromosomes based on segmentally duplicated genes.

Tetrad 1	chr 1	chr 2	chr 3	chr 4
chr 1	NA	0.032	0.051	0.036
chr 2	0.032	NA	0.052	0.036
chr 3	0.051	0.052	NA	0.052
chr 4	0.036	0.036	0.052	NA

Tetrad 2	chr 5	chr 6	chr 7	chr 8
chr 5	2.239	0.033	0.035	0.045
chr 6	0.033	1.988	0.033	0.044
chr 7	0.035	0.033	2.496	0.044
chr 8	0.045	0.044	0.044	2.069

Tetrad 3	chr 9	chr 10	chr 11	chr 12
chr 9	3.254	0.036	0.051	0.035
chr 10	0.036	2.181	0.051	0.035
chr 11	0.051	0.051	2.512	0.05
chr 12	0.035	0.035	0.05	1.903

	chr 13	chr 14	chr 15	chr 16
chr 13	NA	0.05	0.034	0.034
chr 14	0.05	NA	0.049	0.049
chr 15	0.034	0.049	NA	0.03
chr 16	0.034	0.049	0.03	NA

	chr 17	chr 18	chr 19	chr 20
chr 17	NA	0.036	0.05	0.032
chr 18	0.036	NA	0.048	0.034

chr 19	0.05	0.048	NA	0.049
chr 20	0.032	0.034	0.049	NA

	chr 21	chr 22	chr 23	chr 24
chr 21	NA	0.035	0.033	0.047
chr 22	0.035	NA	0.034	0.048
chr 23	0.033	0.034	NA	0.048
chr 24	0.047	0.048	0.048	NA

	chr 25	chr 26	chr 27	chr 28
chr 25	1.875	0.049	0.046	0.042
chr 26	0.049	2.006	0.041	0.042
chr 27	0.046	0.041	2.297	0.035
chr 28	0.042	0.042	0.035	1.421
